# Evolutionarily stable disequilibrium: endless dynamics of evolution in a stationary population

**DOI:** 10.1098/rspb.2015.3109

**Published:** 2016-05-11

**Authors:** Nobuto Takeuchi, Kunihiko Kaneko, Paulien Hogeweg

**Affiliations:** 1 Department of Basic Science, Graduate School of Arts and Sciences, University of Tokyo, Tokyo, Japan; 2 Theoretical Biology and Bioinformatics Group, Utrecht University, Utrecht, The Netherlands

**Keywords:** multilevel selection, prebiotic evolution, intragenomic conflict, RNA world, major evolutionary transition

## Abstract

Evolution is often conceived as changes in the properties of a population over generations. Does this notion exhaust the possible dynamics of evolution? Life is hierarchically organized, and evolution can operate at multiple levels with conflicting tendencies. Using a minimal model of such conflicting multilevel evolution, we demonstrate the possibility of a novel mode of evolution that challenges the above notion: individuals ceaselessly modify their genetically inherited phenotype and fitness along their lines of descent, without involving apparent changes in the properties of the population. The model assumes a population of primitive cells (protocells, for short), each containing a population of replicating catalytic molecules. Protocells are selected towards maximizing the catalytic activity of internal molecules, whereas molecules tend to evolve towards minimizing it in order to maximize their relative fitness within a protocell. These conflicting evolutionary tendencies at different levels and genetic drift drive the lineages of protocells to oscillate endlessly between high and low intracellular catalytic activity, i.e. high and low fitness, along their lines of descent. This oscillation, however, occurs independently in different lineages, so that the population as a whole appears stationary. Therefore, ongoing evolution can be hidden behind an apparently stationary population owing to conflicting multilevel evolution.

## 1. Introduction

Evolution is often conceived as changes in the properties of a population over generations [1–3]. When different forces of evolution are constant in space and time, these properties eventually reach equilibrium, a well-known example being the mutation-selection balance. According to the above notion of evolution, no evolutionary changes are expected to occur at such equilibrium, except random fluctuations due to genetic drift. Although this notion is likely to be valid under many circumstances, does it exhaust the possible dynamics of evolution?

The answer might be ‘no’ as suggested by the following consideration. Life is structured in a hierarchical manner [4–6]. Evolution can operate at multiple levels of hierarchy, and evolutionary tendencies at different levels can be in conflict with one another. For example, the genome of a cell consists of a number of genes. Cells tend to evolve toward ensuring the survival of the cells, whereas genes, toward ensuring the survival of the genes. The former is evident from the evolution of cell-level function such as metabolism; the latter manifests itself in the evolution of selfish genetic elements such as transposons [7,8]. Similar examples abound throughout the biological hierarchy: the evolution of eukaryotes and selfish organelles [9,10], the evolution of multicellular organisms and cancer cells [11], and the evolution of social insects and cheating individuals [12]. Such conflicting multilevel evolution might substantially increase the complexity of evolutionary dynamics even if different forces of evolution are constant in space and time, thereby potentially rendering the above notion of evolution inadequate [13–15].

This consideration led us to investigate a minimal model of conflicting multilevel evolution, taking protocells as the simplest paradigm of hierarchically structured evolving systems [16–18]. Using the model, we show that conflicting multilevel evolution can lead to a novel class of evolutionary dynamics, in which ongoing evolution is hidden behind an apparently stationary population.

## 2. Model

### (a) General description of the model

**Figure 1.**
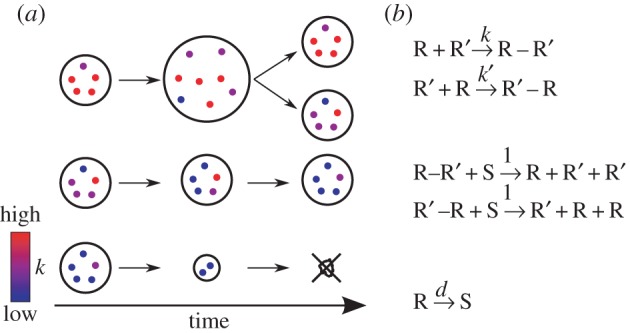
Schematic of the model. (*a*) Protocells containing replicating molecules (substrates are not shown). Colours indicate the catalytic activity of molecules *k*. A protocell with high-*k* molecules grows and divides (top) and that with low-*k* molecules shrinks and dies (bottom). Molecules within a protocell evolve toward decreasing *k* (middle). (*b*) Reaction scheme. R (R′): replicating molecules, R–R′ (R′–R): complex, S: substrate. Each replicating molecule is assigned a unique complex formation rate 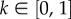. Any pair of molecules can form a complex. Each pair can form two distinct complexes depending on which molecule serves as a catalyst or template, as indicated by prime symbols (top). The complex formation rate is given by the *k* value of the catalyst. Replication produces a new molecule, whose *k* value is copied from the template (middle). This *k* value is slightly modified by a mutation with a probability *m* per replication (see Model). The *k* values of all molecules were initially set to unity at the beginning of each simulation. All molecules decay at the rate *d* (bottom). *m* = 0.01 and *d* = 0.02 unless otherwise stated. (Online version in colour.)

The model assumes a population of protocells, each containing a population of replicating molecules (figure 1*a*; see the next section for details). These molecules can serve both as catalysts and templates for replication. Molecules within a protocell were assumed to have very similar sequences, so that the template specificity of catalysts is ignorable. Thus, a pair of molecules, one serving as a catalyst and the other as a template, form a complex at a catalyst-dependent rate *k* (figure 1*b*, top). Subsequently, the complex converts a substrate into a copy of the template and dissociates (figure 1*b*, middle). This time lag between complex formation and replication, combined with a molecule's finite lifetime, results in a trade-off: if a molecule spends more time serving as a catalyst, it necessarily gets less time to serve as a template, inhibiting its own replication [19]. Consequently, molecules tend to evolve towards minimizing their catalytic activity *k* in order to maximize their relative chance of replication within a protocell—the evolution of selfish templates [7,8,20]. This tendency, if unchecked, would completely halt the replication of molecules within a protocell.

This evolutionary tendency at the molecular level, however, is counteracted by selection between protocells [21]. A protocell was assumed to divide into two when the number of its internal molecules and substrates, hereafter referred to as the cell size, exceeded a threshold value of *V* (the threshold for cell division). The molecules were randomly distributed among the daughter cells. In order to grow and divide, protocells must compete for finite substrates. Substrates are added when replicating molecules decay so that the total number of molecules and substrates is kept constant (this was implemented by assuming the reaction R → S). Substrates diffuse passively across protocells, whereas replicating molecules do not (see the next section). Therefore, a protocell with faster replicating molecules has an advantage because the consumption of substrates induces a net influx of substrates through passive diffusion [22]. Consequently, protocells tend to evolve toward maximizing the catalytic activity of intracellular molecules, counteracting the evolutionary tendency of molecules within each protocell.

### (b) Details of the model

The model consists of a fixed number *N* of replicating molecules and substrates (hereafter referred to as particles for short). Particles are partitioned into protocells, whose number can vary over time. *N* was set to 500 *V*, so that the number of protocells was independent of *V* (under this condition, the number of protocells fluctuated around 2*N*/*V*, i.e. 1 000).

One time step of the model consists of three steps: the reaction, diffusion, and cell-division steps. Each step is described below.

The reaction step consists of *N*/*α* iterations of a reaction algorithm, where *α* is a scaling constant [19]. The reaction algorithm randomly chooses one of *N* particles (denoted by M_1_) with an equal probability. Subsequently, the algorithm randomly chooses a second particle (denoted by M_2_) from the same protocell as that containing M_1_. Depending on M_1_ and M_2_, three types of reactions are possible:

— If both M_1_ and M_2_ are replicating molecules that are not forming any complexes, they can form a complex. Two kinds of complexes are possible depending on which molecule serves as a catalyst or template. Complex formation in which M_1_ serves as a catalyst and M_2_ as a template occurs with a probability *αβk*_1_, where *k*_1_ is the complex formation rate of M_1_ (*β* is described below). Complex formation in which M_2_ serves as a catalyst and M_1_ as a template occurs with a probability *αβk*_2_, where *k*_2_ is the complex formation rate of M_2_. Note that these probabilities are independent of molecules serving as templates (i.e. the template specificity of catalysts was ignored).
— If either M_1_ or M_2_ is forming a complex and the other is a substrate, replication occurs with a probability *αγ* (*γ* is described below). Replication converts a substrate into a copy of the template molecule with a possible mutation (see below).
— If M_1_ is a replicating molecule (whether or not it is forming a complex), M_1_ decays into a substrate with a probability *αd*. If M_1_ is forming a complex, the complex is dissociated before the decay. (M_2_ does not decay.)

Only one of the above reactions occurs with the given probability. In order to ensure that the relative frequencies of these reactions are proportional to their rate constants (namely, *k*_1_, *k*_2_, 1, and *d*), the values of *α*, *β*, and *γ* were chosen as follows. The value of *α* was set such that the sum of the above probabilities never exceeded unity: 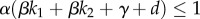. *β* was set to 1/2 because two molecules can be chosen in two different orders. Likewise, *γ* was set to 1/4 because a complex and a substrate can be chosen in two different orders, and a complex has twice the chance of being chosen (because it consists of two molecules). The above reaction algorithm was iterated *N*/*α* times per time step so that the time is independent of *α* and *N*. The above algorithm produces basically the same dynamics as that of the Gillespie algorithm [23] if molecules are not partitioned into protocells [19].

When a new molecule is produced through replication, its *k* value is copied from the template molecule with a possible mutation. The *k* value is mutated with a probability *m* per replication by adding a small number 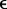 that is uniformly distributed in (−0.05, 0.05). *k* was bounded above by one with a reflecting boundary. *k* was allowed to assume a negative value in order to remove the boundary effect at *k* = 0. When *k* < 0, the rate of complex formation was, however, regarded as zero.

In the diffusion step, all substrates are randomly redistributed among protocells with probabilities proportional to the number of replicating molecules in each protocell (thus, the numbers of substrates within protocells follow a multinomial distribution after a diffusion step). In other words, substrates were assumed to diffuse across protocells extremely quickly compared with the reaction step (this assumption can be relaxed without qualitatively affecting the results as shown in electronic supplementary material, figure S5*a*). By contrast, replicating molecules were assumed not to diffuse at all. This difference in diffusion allows some protocells to outgrow the others by converting substrates into replicating molecules at faster rates (i.e. having higher *k*).

In the cell-division step, every protocell that has more than *V* particles is divided into two, with its particles randomly distributed among the daughter cells. Protocells with no particles are removed. All simulations were run for greater than or equal to 10^7^ time steps unless otherwise stated. A source code implementing the above model is available from Dryad (see Data accessibility).

## 3. Results

### (a) Conflicting multilevel evolution

**Figure 2.**
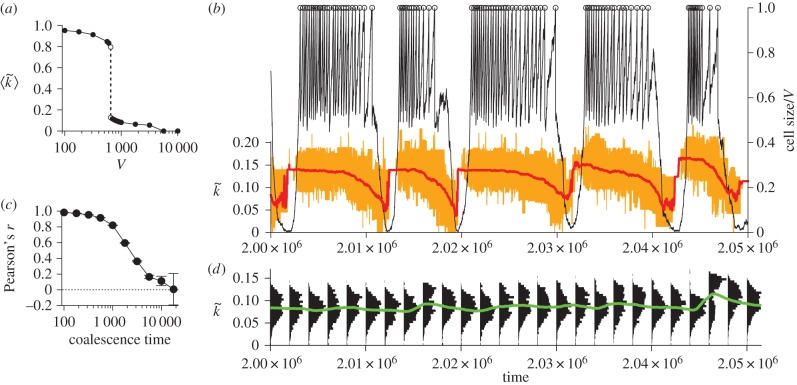
Evolutionary oscillation. (*a*) The average intracellular catalytic activity of protocells (denoted by 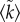) as a function of *V*. The intracellular catalytic activity of a protocell (denoted by 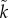) was defined as the average *k* of its internal molecules. When protocells went extinct, 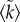 is zero. (*b*) The dynamics of a protocell lineage along its line of descent for *V* = 1 000. The displayed lineage refers to the common ancestors of a population at time 2.5 × 10^6^. Colour coding: normalized cell sizes (black); cell division (circle); 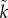 (red); the ranges of *k* of internal molecules (orange). (*c*) The correlation coefficient of 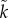 between protocells as a function of coalescence time (error bars, 99% CI; see Methods). (*d*) The frequency distributions of 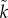 (black), and its average 〈*k*〉 (green) in protocell populations. (Online version in colour.)

The relative strengths of the opposing evolutionary tendencies at the molecular and cellular levels depend on the parameters. For example, *V* determines the average number of molecules per protocell. Decreasing *V*, therefore, increases stochasticity in the evolutionary dynamics of molecules within a protocell. This enhances the effect of random genetic drift and, commensurately, reduces the effect of selection between molecules within a protocell [21]. Moreover, decreasing *V* decreases mutational input per protocell, so that it decelerates the evolution of molecules within protocells [24]. Finally, decreasing *V* increases variation between protocells because it increases the chance of uneven cell division [21]. All these effects strengthen the evolutionary tendency at the cellular level relative to that at the molecular level. Therefore, if *V* is sufficiently small (*V* < 650), the cellular-level evolutionary tendency dominates over the molecular-level evolutionary tendency. In this case, the average intracellular catalytic activity is maximized (figure 2*a*). By contrast, if *V* is sufficiently large (*V* > 5 600), the molecular-level evolutionary tendency dominates over the cellular-level evolutionary tendency. In this case, the average intracellular catalytic activity is minimized, resulting in the extinction of protocells (figure 2*a*). For an intermediate range of *V* (650 < *V* < 5 600), the two evolutionary tendencies are comparable in strength—the situation where conflicting multilevel evolution ensues. In this range of *V*, the two tendencies still balance out, resulting in the stationary frequency distribution of intracellular catalytic activity in the population of protocells (figure 2*d*).

### (b) Evolutionary oscillation

Despite this apparent statistical stasis, protocells ceaselessly change in phenotype and fitness through evolution. Tracking the lineages of protocells revealed that the common ancestors of a population constantly oscillate between two distinct phases—a growing and a shrinking phase—along their line of descent (figure 2*b*). The growing phase is characterized by a rapid increase in the cell size due to the replication of molecules and an abrupt decrease due to cell division. The growing phase is followed by the shrinking phase, which is characterized by a steady decrease in the cell size. The shrinking phase, however, is ended by the sudden resurgence of growth—and the cycle repeats itself. In sync with this phase cycle, internal molecules also oscillate between high and low catalytic activity (figure 2*b*). The catalytic activity decreases during the growing and shrinking phases, but abruptly increases before the revival of cell growth (figure 2*b*). This oscillation of phases and catalytic activity occurs, not only in the common ancestors of a population, but also in all surviving lineages (electronic supplementary material, figure S1). The periods of oscillation are statistically distributed around a single peak (at about 7 000 time steps for *V* = 1 000; electronic supplementary material, figure S2). Thus, the oscillation gets increasingly desynchronized between different lineages with their coalescence time (figure 2*c*). Consequently, the frequency distribution of intracellular catalytic activity—i.e. a phenotype of protocells—appears stationary (figure 2*d*). Nevertheless, protocells are in perpetual, regular evolutionary motion in phenotype and therefore in fitness along their lines of descent. Hence, survival of the fittest does not hold.

This evolutionary oscillation, although unexpected, has a simple explanation based on conflicting multilevel evolution and population bottlenecks. To see this, consider the dynamics of a lineage of protocells along its line of descent. As a protocell grows and divides, its intracellular catalytic activity inevitably decreases owing to the evolution of internal molecules. Eventually, the protocell is put at a disadvantage for substrate competition, and its internal molecules start to decrease in number. In the majority of cases, all the molecules eventually decay, and the protocell dies. In rare cases, however, molecules with high catalytic activity survive through genetic drift induced by a severe population bottleneck. In this case, the protocell regains a competitive advantage and can grow again, starting another cycle of the evolutionary oscillation. Although protocells rarely succeed in the resurgence of growth (a probability approximately 10^−2^), only those that succeed can survive and proliferate because of between-protocell competition. Therefore, in all surviving lineages (i.e. all observable lineages), protocells always undergo resurgence after shrinking (figure 2*b*; electronic supplementary material, figure S1). To sum up, the evolutionary oscillation is due to the dynamic interplay between conflicting multilevel evolution and intracellular population bottlenecks.

**Figure 3.**
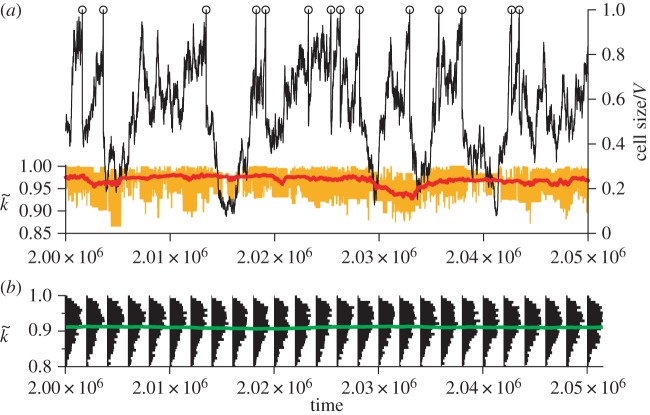
No evolutionary oscillation in the absence of conflicting multilevel evolution. (*a*) The dynamics of a protocell lineage along its line of descent for *V* = 316. The displayed lineage refers to the common ancestors of a population at time 2.5×10^6^. Colour coding as in figure 2*b*. (*b*) The frequency distributions of 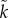 (black), and its average 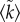 (green) in protocell populations. (Online version in colour.)

**Figure 4.**
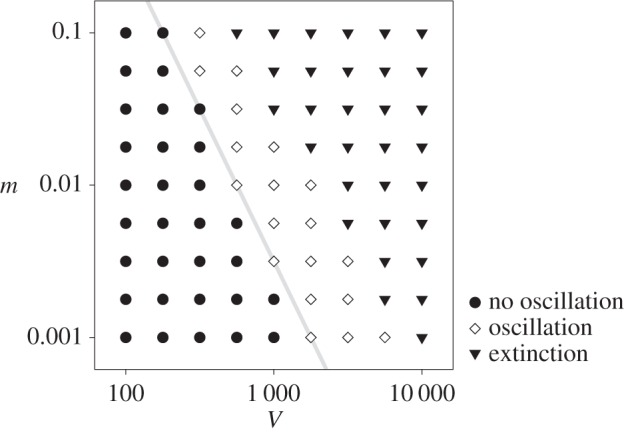
Phase diagram with respect to *V* and *m*. The boundaries between the parameter regions where the evolutionary oscillation occurs (diamond) and where it does not occur (circle) has approximately the same slope as that of *mV*^2^ = const. (grey line); extinction (triangle). See Methods for the details of the computation and parameters.

According to the above explanation, the evolutionary oscillation should cease to operate if the conflict of multilevel evolution is reduced. To verify this expectation, we increased the evolutionary tendency at the cellular level by decreasing the value of *V*. When *V* is sufficiently small (*V* < 650), the evolutionary oscillation ceases to operate, as expected (figure 3). We also carried out the same analysis with respect to *m*, the mutation rate of replicating molecules. Decreasing *m* decreases the mutational input per protocell, so that it decreases the evolutionary tendency at the molecular level. Therefore, the evolutionary oscillation was expected only for an intermediate range of *m* for a fixed value of *V*, an expectation confirmed in electronic supplementary material, figure S3. Moreover, this range of *m* should shift to smaller values as *V* increases. This is also confirmed by a phase diagram displaying the parameter region in which the evolutionary oscillation occurs (figure 4). The phase diagram also reveals an approximate scaling-relationship, *mV*^2^ = constant (const.), for a boundary between the parameter regions where the oscillation occurs and where no oscillation occurs without extinction. This relationship can be interpreted as follows. The average *k* value within a protocell (denoted by 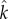) tends to decrease through the evolution of internal molecules. The rate of this decrease should be proportional to the number of mutations occurring per protocell per unit time, according to population genetics, and therefore to *mV*. This decrease, however, is counteracted by selection between protocells, which tends to increase the average value of 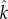 among protocells. The rate of this increase is proportional to the variance of 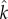 among protocells according to Fisher's fundamental theorem of natural selection [25]. If we assume that this variance is proportional to 1/*V*, we obtain the scaling relationship *mV* ∼ 1/*V* by supposing that the two rates are comparable to each other, the condition under which the evolutionary oscillation is expected. Taken together, the above results support the statement that the evolutionary oscillation is due to conflicting multilevel evolution.

In addition, we varied the decay rate of molecules and the diffusion rate of substrates, and also allowed for complex dissociation. We confirmed that the evolutionary oscillation occurs under a wide range of conditions (electronic supplementary material, figures S4–S6).

### (c) Function of evolutionary oscillation

**Figure 5.**
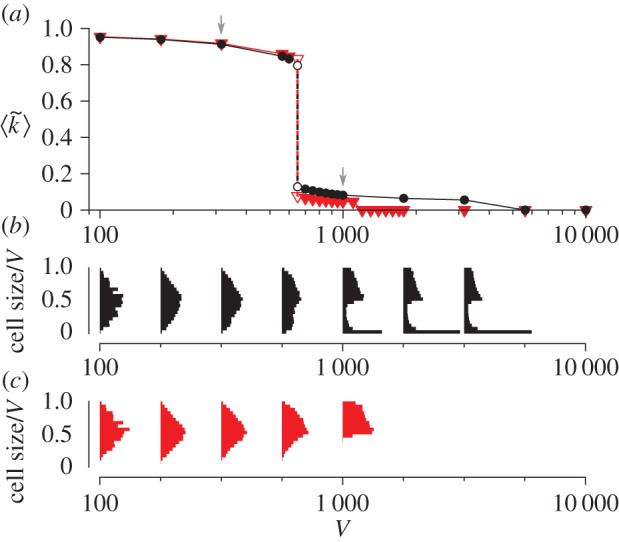
The effect of preventing the evolutionary oscillation. (*a*) The average intracellular catalytic activity as a function of *V*. Symbols: the original model (circle), the model in which the evolutionary oscillation was prevented by killing small protocells (triangle). The arrows indicate *V* = 1 000 (used in figure 2*b–d*) and *V* = 316 (used in figure 3). Unfilled symbols indicate meta-stable states. When protocells went extinct, 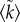 is zero. (*b,c*) The frequency distributions of the cell sizes (scaled by *V*) of common ancestors as a function of *V*. The original model (*b*). The model in which the evolutionary oscillation was prevented (*c*). The bimodality of distributions indicates the occurrence of evolutionary oscillation (*V* ≥ 1 000 in *b*). (Online version in colour.)

We next examined the functional significance of the evolutionary oscillation. To this end, the oscillation was prevented by killing small protocells, i.e. protocells whose cell sizes fell below a threshold of 0.1 *V* (the killing was implemented by converting all internal molecules of a protocell into substrates so that the total number of molecules and substrates was kept constant). This threshold was set much higher than the minimum number of molecules during population bottlenecks, so that the killing prevented the evolutionary oscillation (figure 5*b,c*; electronic supplementary material, figure S7). The killing of small protocells preferentially eliminates protocells with lower intracellular catalytic activity (i.e. lower fitness), so that it reinforces selection between protocells. Therefore, the killing might be expected to increase the average fitness of protocells. However, the killing also prevents molecules from undergoing a population bottleneck, an event that can potentially increase their average catalytic activity through random genetic drift. Therefore, the killing might actually decrease the fitness of protocells, a result that would indicate the functional significance of the evolutionary oscillation. Figure 5*a* shows that the killing, in fact, drives protocells to extinction if *V* > 1 100, reducing the range of *V* for which protocells survive by more than threefold. Moreover, the killing decreases the intracellular catalytic activity by twofold (from about 0.1 to 0.05) for an intermediate range of *V* (650 < *V* < 1 100). By contrast, the killing marginally increases the intracellular catalytic activity for small values of *V* (less than 650), the parameter range in which the evolutionary oscillation does not occur irrespective of the killing. Taken together, the killing of small, unfit protocells substantially decreases the fitness of protocells for sufficiently large *V* (greater than 650). This result is diametrically opposite to the simple expectation based on natural selection and indicates that the evolutionary oscillation is beneficial to the stable maintenance of intracellular catalytic activity. More specifically, this beneficial effect stems from intracellular population bottlenecks, which can neutralize the evolutionary tendency at the molecular level.

Note, however, that this beneficial effect of population bottlenecks is not the reason why these bottlenecks occur. Rather, they occur as a by-product of evolution within protocells, which reduces intracellular catalytic activity, and competition between protocells, which causes protocells with low intracellular catalytic activity to shrink. These bottlenecks, in turn, increase competition between protocells because they can generate protocells with high intracellular catalytic activity. This feedback between molecular and cellular evolutionary dynamics provides stability to the catalytic activity of the entire system and also causes the evolutionary oscillation. This cross-hierarchical feedback is the novel feature of the present model that is absent from the previous models of multilevel selection [26–29].

## 4. Discussion

Life is hierarchically structured, so that evolution can operate at multiple levels with conflicting tendencies. To understand the consequences of such conflicting multilevel evolution, we investigated a model of protocells as the simplest paradigm of hierarchically structured evolving systems. The results described above indicate that conflicting multilevel evolution with cross-hierarchical feedback can lead to a novel class of evolutionary dynamics, the evolutionary oscillation, in which lineages ceaselessly modify their genetically inherited phenotype along their lines of descent, even though the statistical properties of the whole population appear stationary over generations. This oscillation differs from the previously known biological oscillation such as the cell cycle, predator–prey cycle, rock–paper–scissors cycle [30–32], and flush–crash cycle [33]. Whereas the latter involve only processes at a single level (individual or population) and are directly observable at that level, the former requires feedback between evolution at multiple levels and is observable only through lineage tracking. Note also that selection between protocells is frequency-independent (unlike in the rock–paper–scissors game).

Such perpetual evolutionary motion hidden behind an apparently stationary population might be termed *evolutionarily stable disequilibrium*. Evolutionarily stable disequilibrium illustrates a potential discrepancy between evolution and its textbook definition, i.e. changes in the properties of a population, such as the frequency distribution of different genotypes, over generations [1–3]. This discrepancy stems from the fact that if evolution operates at multiple levels, a higher-level entity contains an evolving population of lower-level entities, so that its genetic make-up undergoes not only random changes due to mutation, but also non-random changes due to evolution at the lower level, with resulting feedback to evolution at the higher level. Under this mode of evolution, the detection of ongoing evolution might require the tracking of individual lineages as demonstrated above, a measurement that is becoming feasible in the laboratory [34,35]. Multilevel evolution therefore necessitates expanding our notion of evolution by considering not only the dynamics of populations, but also the dynamics of lineages and cross-hierarchical feedback.

The prevalence of evolutionarily stable disequilibrium in nature is an open question. The general implication of the work presented above is that evolutionarily stable disequilibrium can occur when organisms are subject to conflicting multilevel evolution. In the case of protocells, such a situation might arise if protocells must contain (or exchange) a large number of molecules in order to divide (or maintain high catalytic diversity) or when mutation rates are high during the early evolution. For cases beyond protocells, organisms potentially subject to conflicting multilevel evolution include those containing independently replicating symbionts [36] or genetic elements [7,8], viruses undergoing collective transmission [37], social groups multiplying through fission [38], and organisms immediately after any major evolutionary transition [4,5].

## 5. Methods

### (a) Measurement of the correlation coefficients between lineages

The data shown in figure 2*c* were obtained as follows. The lineages of all protocells, including those that died, were tracked. For every coalescence event between the lineages, the intracellular catalytic activities 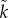 of the coalescing lineages were recorded as a function of time since coalescence. Subsequently, the lineage with a shorter surviving time was removed from the data in order to prevent data redundancy (in the case of a tie, a randomly chosen lineage was removed). Therefore, each coalescence event gave a pair of 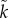 values as a function of coalescence time. The Pearson correlation coefficients were calculated from all these pairs at different coalescence times. The confidence intervals were calculated using Fisher's *z*-transformation.

### (b) Computation of the phase diagram

The data shown in figure 4 were obtained as follows. The presence or absence of the evolutionary oscillation was inferred from sudden changes in the equilibrium value of 〈*k̂*〉 as a function of *V* and *m*. This inference was subsequently confirmed by lineage tracking for several parameter combinations near the boundaries between the different phases. To speed up the computation, the system size and duration of simulations were decreased by fivefold (namely, *N* = 100 *V* instead of 500 *V*, and greater than or equal to 2 × 10^6^ time steps instead of greater than or equal to 10^7^). Decreasing the system size shifts the boundaries between the phases to smaller values of *V* and *m*.

## Supplementary Material

Supplementary figures

## Data accessibility

C++ source code implementing the model: Dryad: http://dx.doi.org/10.5061/dryad.c5t22.
