# Supplementary material for: Evolutionarily stable disequilibrium: endless dynamics of evolution in a stationary population
Source: Proc Biol Sci. 2016 May 11;283(1830):20153109. doi: 10.1098/rspb.2015.3109 (PMC4874703; doi:10.1098/rspb.2015.3109)
Supplement: Supplementary figures [file rspb20153109supp1.pdf]

Supplementary material

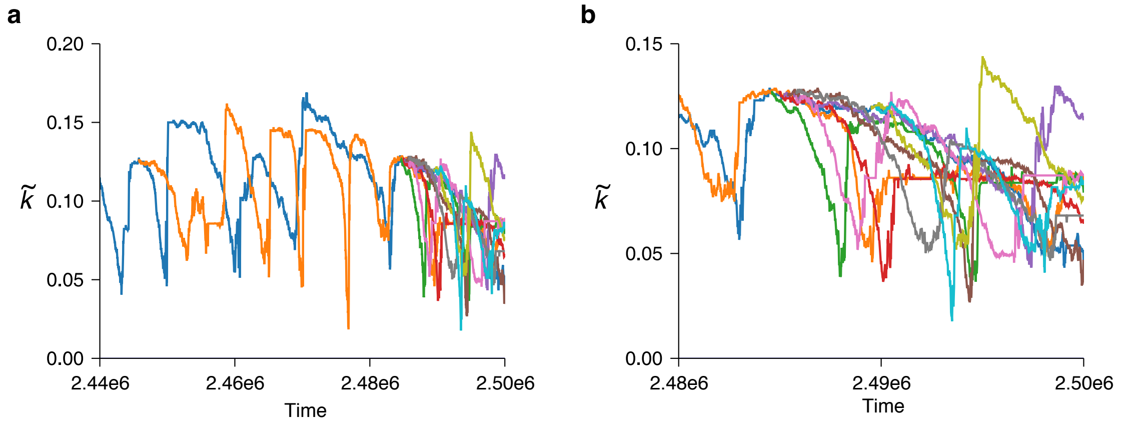

Figure S1: **Evolutionary oscillation in all surviving protocell lineages.** The intracellular catalytic activity $\tilde{k}$ of ten lineages are shown. The ten lineages were chosen from the population at time 2.5×10^6 such that the coalescence events between them are the deepest (i.e., oldest) among those between all individuals in the population. Panels a and b differ only in terms of time window. As this figure shows, all surviving lineages undergo evolutionary oscillation. The data were taken from the same simulation as shown in Fig. 2b.

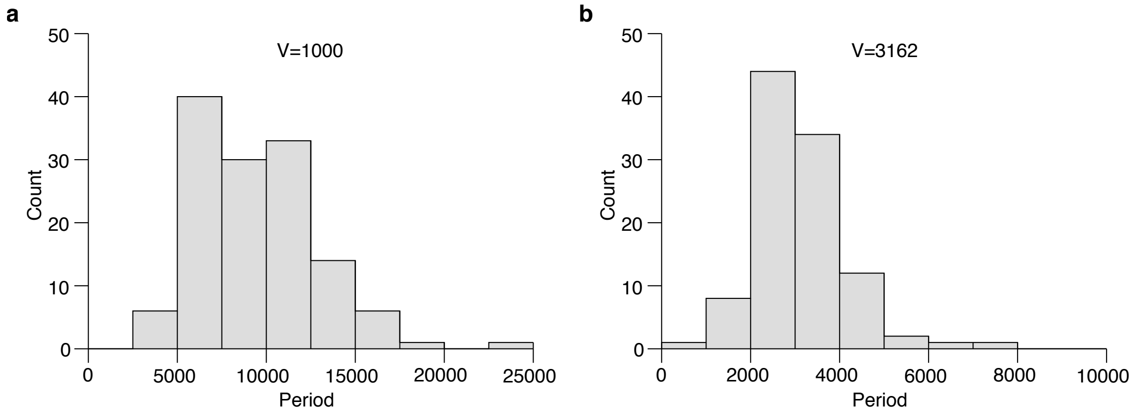

Figure S2: **The frequency distributions of the periods of oscillation. a.** $V = 1000$. The mean, 9353 ($\pm$ 3320 SD). **b.** $V = 3162$. The mean, 3142 ($\pm$ 986 SD). [The details of the measurement: The period of oscillation was defined as the sum of the durations of one growing phase and one shrinking phase that follows. A protocell was considered to transition into a shrinking phase if its size decreased below $0.2V$. Likewise, a protocell was considered to transition into a growing phase if its size exceeded $0.7V$ (and if it was previously in a shrinking phase). To increase the sample size, the periods were sampled from the lineages of all protocells that existed between 2×10^6 and 2.5×10^6 time steps including those that died. To avoid data redundancy, the entire lineage tree was broken up into lineal branches at every coalescent point by detaching, from the main tree, the lineage with shorter surviving time (in the case of tie, a randomly chosen lineage was detached). For every lineal branch, the periods of oscillation were sampled. Note that the total count of periods is greater for $V = 1000$ (131) than for $V = 3162$ (103), even though the mean period is greater for $V = 1000$. This apparent inconsistency is due to the fact that the number of coexisting lineages decreases with V.]

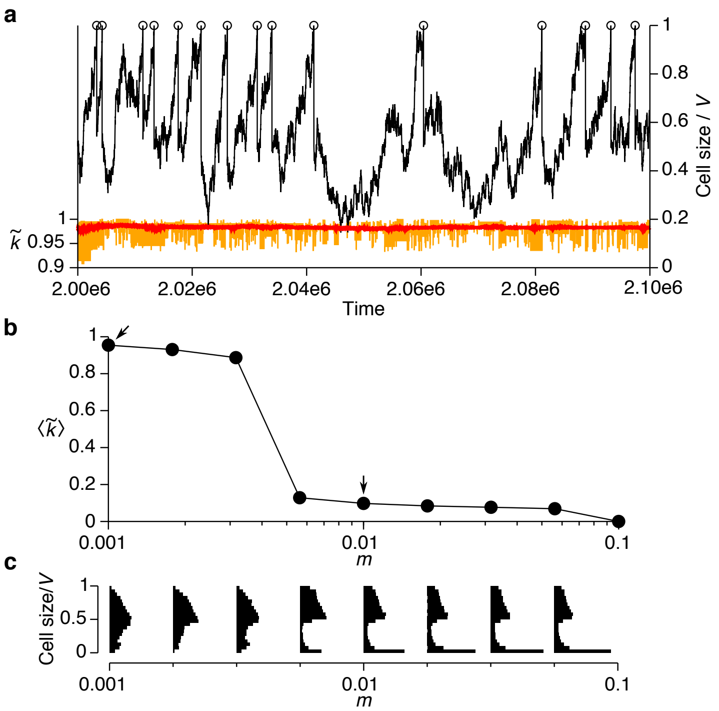

Figure S3: **Parameter sweep with respect to the mutation rate m.** $V = 1000$. **a.** The absence of evolutionary oscillation for a small mutation rate ($m = 0.001$). **b.** The average intracellular catalytic activity as a function of m. The arrows indicate $m = 0.001$ (used in Panel a) and $m = 0.01$ (used in Fig. 2). Protocells go extinct for $m \geqslant 0.1$. **c.** The frequency distribution of cell sizes (scaled by V) of common ancestors as a function of m. The bimodality of distributions indicates the occurrence of evolutionary oscillation ($m \geqslant 0.0056$).

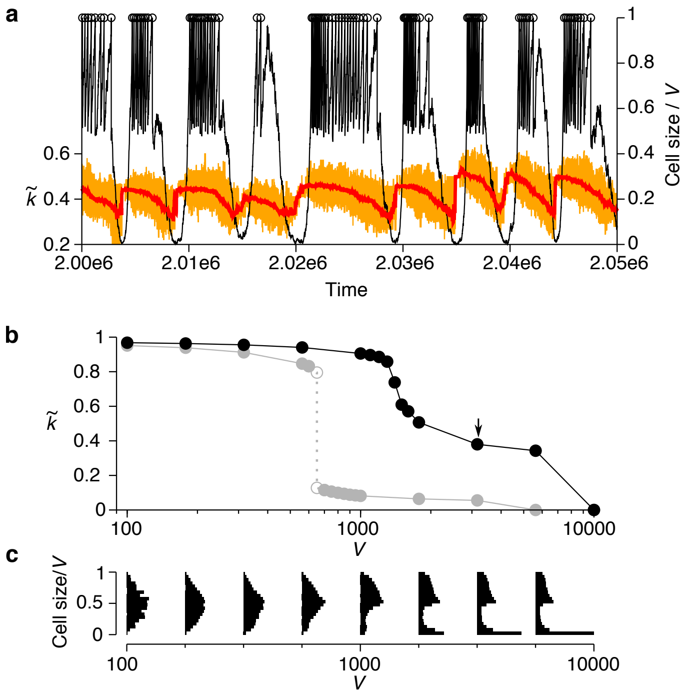

Figure S4: **Evolutionary oscillation for a high decay rate** ($d = 0.0632$). **a.** The dynamics of a protocell lineage along its line of descent ($V = 3162$). The displayed lineage was the common ancestors of a population at time 2.5×10^6. Color coding as in Fig. 2b. **b.** The average intracellular catalytic activity as a function of V for $d = 0.0632$ (•). The arrow indicates $V = 3162$ (used in Panel a). For comparison, the data for $d = 0.02$ (•) is also shown (the same data as in Figs. 2b and 5a). **c.** The frequency distributions of the cell sizes (scaled by V) of common ancestors as a function of V. The bimodality of distributions indicates the occurrence of evolutionary oscillation ($V \geqslant 1778$).

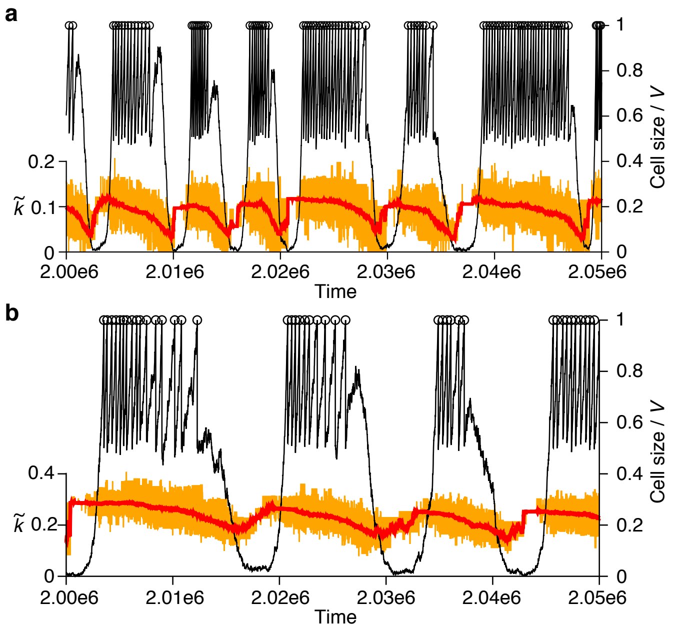

Figure S5: **Evolutionary oscillation under various conditions. a.** Evolutionary oscillation in the model with finite diffusion ($V = 1000$). The diffusion step in this model consists of DN iterations of a diffusion algorithm, where D is a diffusion rate, and N is the total number of replicating molecules and substrates (collectively, particles, for short). The diffusion algorithm randomly chooses two particles from all protocells. If the two particles are a replicating molecule and a substrate, the substrate is transferred to the protocell that contains the replicating molecule. The results shown in this figure were obtained with a small diffusion rate $D = 0.1$ (if $D = 1$, one single diffusion step more or less completely randomizes the distribution of substrates over protocells). Color coding as in Fig. 2b. The displayed lineage was the common ancestors of a population at time 2.5×10^6. **b.** Evolutionary oscillation in the model with complex dissociation ($V = 1778$). All complexes dissociate at a constant rate 0.2. Color coding as in Fig. 2b. The displayed lineage was the common ancestors of a population at time 2.5×10^6.

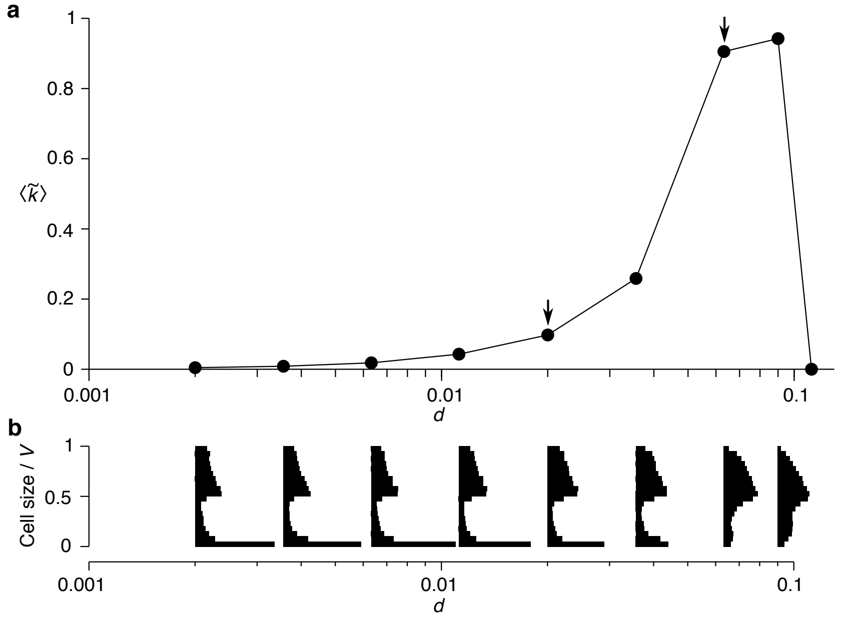

Figure S6: **Parameter sweep with respect to the decay rate d for $V = 1000$. a.** The average intracellular catalytic activity as a function of d. The arrows indicate $d = 0.02$ (used in Fig. 2) and $d = 0.0632$ (used in Fig. S4). Protocells go extinct for $d \geqslant 0.11$. **b.** The frequency distribution of cell sizes (scaled by V) of common ancestors as a function of d. The bimodality of distributions indicates the occurrence of evolutionary oscillation ($d \leqslant 0.0356$).

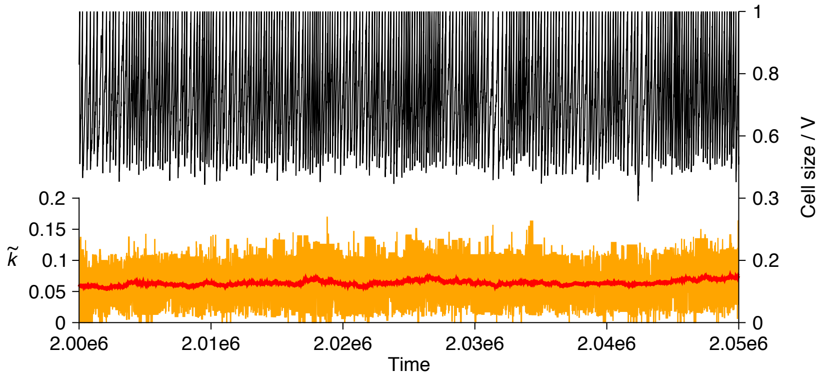

Figure S7: **The absence of evolutionary oscillation in the model in which small protocells were killed.** The dynamics of a protocell lineage along its line of descent ($V = 1000$). The displayed lineage was the common ancestors of a population at time 2.5×10^6. Color coding as in Fig. 2b. Cell division is not marked for visibility.
